# Colchicine in patients admitted to hospital with COVID-19 (RECOVERY): a randomised, controlled, open-label, platform trial

**DOI:** 10.1016/S2213-2600(21)00435-5

**Published:** 2021-12

**Authors:** RECOVERY Collaborative Group

## Abstract

**Background:**

Colchicine has been proposed as a treatment for COVID-19 based on its anti-inflammatory actions. We aimed to evaluate the efficacy and safety of colchicine in patients admitted to hospital with COVID-19.

**Methods:**

In this streamlined, randomised, controlled, open-label trial, underway at 177 hospitals in the UK, two hospitals in Indonesia, and two hospitals in Nepal, several possible treatments were compared with usual care in patients hospitalised with COVID-19. Patients were eligible for inclusion in the study if they were admitted to hospital with clinically suspected or laboratory confirmed SARS-CoV-2 infection and had no medical history that might, in the opinion of the attending clinician, put the patient at significant risk if they were to participate in the trial. Eligible and consenting adults were randomly assigned (1:1) to receive either usual standard of care alone (usual care group) or usual standard of care plus colchicine (colchicine group) using web-based simple (unstratified) randomisation with allocation concealment. Participants received colchicine 1 mg after randomisation followed by 500 μg 12 h later and then 500 μg twice a day by mouth or nasogastric tube for 10 days in total or until discharge. Dose frequency was halved for patients receiving a moderate CYP3A4 inhibitor (eg, diltiazem), patients with an estimated glomerular filtration rate of less than 30 mL/min per 1·73m^2^, and those with an estimated bodyweight of less than 70 kg. The primary outcome was 28-day mortality, secondary endpoints included time to discharge, the proportion of patients discharged from hospital within 28 days, and, in patients not on invasive mechanical ventilation at randomisation, a composite endpoint of invasive mechanical ventilation or death. All analyses were by intention-to-treat. The trial is registered with ISRCTN, 50189673, and ClinicalTrials.gov, NCT04381936.

**Findings:**

Between Nov 27, 2020, and March 4, 2021, 11 340 (58%) of 19 423 patients enrolled into the RECOVERY trial were eligible to receive colchicine; 5610 (49%) patients were randomly assigned to the colchicine group and 5730 (51%) to the usual care group. Overall, 1173 (21%) patients in the colchicine group and 1190 (21%) patients in the usual care group died within 28 days (rate ratio 1·01 [95% CI 0·93 to 1·10]; p=0·77). Consistent results were seen in all prespecified subgroups of patients. Median time to discharge alive (10 days [IQR 5 to >28]) was the same in both groups, and there was no significant difference in the proportion of patients discharged from hospital alive within 28 days (3901 [70%] patients in the colchicine group and 4032 [70%] usual care group; rate ratio 0·98 [95% CI 0·94 to 1·03]; p=0·44). In those not on invasive mechanical ventilation at baseline, there was no significant difference in the proportion meeting the composite endpoint of invasive mechanical ventilation or death (1344 [25%] in the colchicine group *vs* 1343 [25%] patients in the usual care group; risk ratio 1·02 [95% CI 0·96 to 1·09]; p=0·47).

**Interpretation:**

In adults hospitalised with COVID-19, colchicine was not associated with reductions in 28-day mortality, duration of hospital stay, or risk of progressing to invasive mechanical ventilation or death.

**Funding:**

UK Research and Innovation (Medical Research Council), National Institute of Health Research, and Wellcome Trust.

## Introduction

Inflammation is a key feature of severe COVID-19. Markedly raised concentrations of inflammatory markers, such as C-reactive protein (CRP), ferritin, interleukin-6 (IL-6), and other cytokines, are observed in severe cases and are associated with poor outcomes.^1^, ^2^, ^3^, ^4^, ^5^ Inflammation is particularly prominent in the lung and vascular endothelium, and is commonly associated with extensive alveolar damage and thrombosis of large and small pulmonary vessels.^6^ Corticosteroids and IL-6 inhibitors have both been shown to reduce mortality in patients with severe COVID-19; Janus kinase (JAK) inhibitors accelerate improvement in clinical status.^7^, ^8^, ^9^, ^10^ Together these results show that inflammation is modifiable and anti-inflammatory regimens can improve clinical outcomes.

Inflammasomes are a key part of the innate immune response to SARS-CoV-2 infection. These cytosolic pattern recognition receptor systems are activated in response to detection of pathogens in the cytosol and stimulate the release of proinflammatory cytokines.^11^ In COVID-19, the degree of inflammasome activation, particularly the nucleotide binding domain (NOD)-like pyrin domain 3 (NLRP3) inflammasome, correlates with disease severity.^12^ Colchicine, a readily available, safe, and inexpensive drug, has a wide range of anti-inflammatory effects, including inhibition of the NLRP3 inflammasome.^13^ In addition to its role in treating acute gout and pericarditis, evidence is emerging that colchicine might inhibit endovascular inflammation and provide clinical benefits in patients with coronary artery disease.^14^, ^15^, ^16^, ^17^ Given the activation of NLRP3 in COVID-19 and the presence of vascular endothelial inflammation, colchicine has been proposed as a treatment for SARS-CoV-2 associated inflammatory disease. However, only three small randomised trials have assessed the effects of colchicine in hospitalised patients with COVID-19, with a total of only seven deaths across these studies combined; none of the studies were adequately powered to identify any effect of the drug on mortality.^18^, ^19^, ^20^ Here we report the results of a large randomised controlled trial that aimed to evaluate the efficacy and safety of colchicine in patients hospitalised with COVID-19.

**Research in context**

**Evidence before this study**

We searched medRxiv, bioRxiv, Medline, Embase, and the WHO International Clinical Trials Registry Platform from Sept 1, 2019, to April 1, 2021, for clinical trials evaluating the effect of colchicine treatment in patients hospitalised with COVID-19, using the search terms (“SARS-CoV-2.mp” OR “COVID.mp” OR “COVID-19.mp” OR “2019-nCoV.mp” OR “Coronavirus.mp” OR “Coronavirinae/”) AND (“colchicine.mp” OR “colchicine/”), using validated filters to select for randomised controlled trials. No language restrictions were applied.

We identified three relevant randomised trials that compared colchicine with usual care or placebo in patients hospitalised with COVID-19 (two at low risk of bias and one with some concerns because of limited information on randomisation process and poor clarity regarding the blinding in the study). Each trial suggested a potential favourable effect of colchicine on outcome measures of clinical improvement or duration of hospitalisation. The three trials combined included a total of 285 patients and seven deaths; even combined, these trials were not adequately powered to detect an effect on mortality.

**Added value of this study**

The RECOVERY trial is the first large, randomised trial to report results of the effect of colchicine in patients hospitalised with COVID-19. We found no significant effect of colchicine compared with usual care alone on 28-day mortality, the probability of discharge alive within 28 days, or the probability of progressing to the composite outcome of invasive mechanical ventilation or death in patients who were not receiving invasive mechanical ventilation at randomisation. We saw no evidence of benefit of colchicine in any patient subgroup.

**Implications of all the available evidence**

There is no good evidence that colchicine treatment is of clinical benefit for adults hospitalised with COVID-19 compared with current usual care.

## Methods

### Study design and participants

The RECOVERY trial is an investigator-initiated, streamlined, individually randomised, controlled, open-label, platform trial to evaluate the effects of potential treatments in patients hospitalised with COVID-19. Details of the trial design and results for other possible treatments (dexamethasone,^7^ hydroxychloroquine,^21^ lopinavir–ritonavir,^22^ azithromycin,^23^ tocilizumab,^9^ and convalescent plasma^24^) have been published previously. The trial is underway at 177 hospitals in the UK, two hospitals in Indonesia, and two hospitals in Nepal (appendix pp 3–25). The trial is supported by the National Institute for Health Research Clinical Research Network, and is coordinated by the Nuffield Department of Population Health (University of Oxford, Oxford, UK), the trial sponsor. The trial was done in accordance with the principles of the International Conference on Harmonisation–Good Clinical Practice guidelines and approved by the UK Medicines and Healthcare products Regulatory Agency (MHRA) and the Cambridge East Research Ethics Committee (20/EE/0101). The protocol, statistical analysis plan, and additional information are available online.

Patients admitted to hospital were eligible for the study if they had clinically suspected or laboratory confirmed SARS-CoV-2 infection and no medical history that might, in the opinion of the attending clinician, put the patient at significant risk if they were to participate in the trial. Children and pregnant women were not eligible to receive colchicine. Patients with severe liver impairment, significant cytopaenia, concomitant use of strong CYP3A4 (eg, clarithromycin, erythromycin, systemic azole antifungal, and HIV protease inhibitor) or P-glycoprotein inhibitors (eg, ciclosporin, verapamil, and quinidine), or hypersensitivity to lactose were excluded from the colchicine comparison (appendix p 81). Written informed consent was obtained from all patients, or a legal representative if patients were too unwell or unable to provide consent.

### Randomisation and masking

Baseline data were collected using a web-based case report form that included demographics, level of respiratory support, major comorbidities, suitability of the study treatment for a particular patient, and treatment availability at the study site (appendix pp 32–34). Eligible and consenting, non-pregnant adult patients were randomly assigned (1:1) to receive either usual standard of care (usual care group) or usual standard of care plus colchicine (colchicine group), or one of the other available RECOVERY treatment groups, using web-based simple (unstratified) randomisation with allocation concealed until after randomisation (appendix pp 30–31). For some patients, colchicine was unavailable at the hospital at the time of enrolment or was considered by the managing physician to be either definitely indicated or definitely contraindicated. These patients were excluded from the randomised comparison between colchicine and usual care. Patients received colchicine 1 mg after randomisation followed by 500 μg 12 h later and then 500 μg twice a day orally or by nasogastric tube for 10 days in total or until discharge, whichever occurred first. Dose frequency was halved for patients receiving a moderate CYP3A4 inhibitor (eg, diltiazem), those who had renal impairment (estimated glomerular filtration rate <30 mL/min per 1·73 m^2^), and patients with an estimated bodyweight of less than 70 kg (appendix p 81).

As a platform trial, and in a factorial design, patients could be simultaneously randomly assigned to other treatment groups: 1) convalescent plasma versus casirivimab and imdevimab versus usual care, 2) aspirin versus usual care, and 3) baricitinib versus usual care (appendix p 31). Until Jan 24, 2021, the trial also allowed a subsequent randomisation for patients with progressive COVID-19 (evidence of hypoxia and a hyperinflammatory state) to receive usual care plus tocilizumab or usual care alone. Participants and local study staff were not masked to the allocated treatment. The trial steering committee, investigators, and all other individuals involved in the trial were masked to outcome data during the trial.

### Procedures

A single online follow-up form was completed when participants were discharged, had died, or 28 days after randomisation, whichever occurred first (appendix pp 35–41). Information was recorded on adherence to allocated study treatment, receipt of other COVID-19 treatments, duration of admission, receipt of respiratory or renal support, and vital status (including cause of death). In addition, in the UK, routine health-care and registry data were obtained, including information on vital status (with date and cause of death), discharge from hospital, receipt of respiratory support, or renal replacement therapy.

### Outcomes

Outcomes were assessed 28 days after randomisation, with additional analyses specified at 6 months. The primary outcome was all-cause mortality. Secondary outcomes were time to discharge from hospital alive within 28 days and, in patients not on invasive mechanical ventilation at randomisation, receipt of invasive mechanical ventilation (including extracorporal membrane oxygenation) or death. Prespecified subsidiary clinical outcomes were use of non-invasive respiratory support, time to successful cessation of invasive mechanical ventilation (defined as cessation of invasive mechanical ventilation within, and survival to, 28 days), use of haemodialysis or haemofiltration, cause-specific mortality, bleeding events, thrombotic events, and major cardiac arrhythmias. Information on suspected serious adverse reactions was collected in an expedited fashion to comply with regulatory requirements.

### Statistical analysis

The primary analysis for all outcomes was assessed according to the intention-to-treat principle by comparing patients randomly assigned to the colchicine group with those who were randomly assigned to the usual care group, but for whom colchicine was both available and a suitable treatment. For the primary outcome, all-cause 28-day mortality, the log-rank observed minus expected statistic and its variance were used to test both the null hypothesis of equal survival curves (ie, the log-rank test) and to calculate the one-step estimate of the average mortality rate ratio. We used Kaplan-Meier survival curves to display cumulative mortality over the 28-day period. We used the same method to analyse time to hospital discharge and successful cessation of invasive mechanical ventilation, with patients who died in hospital right-censored on day 29. Median time to discharge was derived from Kaplan-Meier estimates. For the prespecified composite secondary outcome of progression to invasive mechanical ventilation or death within 28 days in those not receiving invasive mechanical ventilation at randomisation, and the subsidiary clinical outcomes of receipt of ventilation and use of haemodialysis or haemofiltration, the precise dates were not available and so the risk ratio was estimated.

Prespecified analyses were done for the primary outcome using the statistical test of interaction (test for heterogeneity or trend), in accordance with the prespecified analysis plan, defined by characteristics at randomisation: age, sex, ethnicity, level of respiratory support, days since symptom onset, and use of corticosteroids (appendix p 115). An exploratory analysis of the primary outcome by CRP concentration at randomisation was done using a similar approach.

Estimates of rate ratios and risk ratios are shown with 95% CIs. All p values are two-sided and are shown without adjustment for multiple testing. The full database is held by the study team, which collected the data from study sites and did the analyses at the Nuffield Department of Population Health, University of Oxford (Oxford, UK).

As stated in the protocol, appropriate sample sizes could not be estimated when the trial was being planned at the start of the COVID-19 pandemic (appendix p 55). As the trial progressed, the trial steering committee, whose members were unaware of the results of the trial comparisons, determined that sufficient patients should be enrolled to provide at least 90% power at a two-sided significance level of 0·01 to detect a clinically relevant proportional reduction in 28-day mortality of 12·5% between the two groups.

On March 4, 2021, the independent data monitoring committee did a routine review of the available safety and efficacy data. The independent data monitoring committee notified the chief investigators that there was no convincing evidence that continued recruitment to the colchicine comparison would provide conclusive proof of worthwhile mortality benefit either overall or in any prespecified subgroup. Consequently, recruitment to the colchicine comparison was closed on March 5, 2021, and preliminary results were made available to the public.

Analyses were done using SAS (version 9.4) and (R version 3.4). The trial is registered with ISRCTN, 50189673, and ClinicalTrials.gov, NCT04381936.

### Role of the funding source

The funder of the study had no role in study design, data collection, data analysis, data interpretation, or writing of the report. The corresponding authors had full access to all the data in the study and had final responsibility for the decision to submit for publication.

## Results

**Figure 1:**
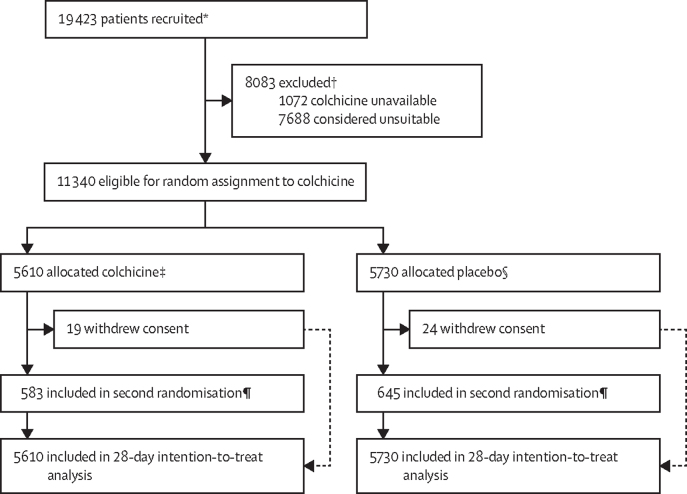
Trial profile Of the 11 340 patients randomly assigned to receive colchicine or usual care, 7091 (63%; 3505 [62%] of the colchicine group *vs* 3586 [63%] of the usual care group) patients were additionally randomised to receive convalescent plasma or REGN–COV2 or usual care; 7545 (67%; 3747 [67%] of the colchicine group *vs* 3798 [66%] of the usual care group) patients were additionally randomised to receive aspirin or usual care; and 1635 (14%; 802 [14%] of the colchicine group vs 833 [15%] of the usual care group) patients were additionally randomised to receive baricitinib or usual care. *Number recruited overall during period that adult participants could be recruited into colchicine comparison. †Colchicine unavailable and colchicine unsuitable groups are not mutually exclusive. ‡5122 (93%) of 5510 patients with completed follow-up at time of analysis received colchicine. §20 (<1%) of 5605 patients with completed follow-up at time of analysis received colchicine. ¶Includes 251 (4%) of 5610 patients in the colchicine group and 306 (5%) of 5730 patients in the usual care group allocated to receive tocilizumab.

Between Nov 27, 2020, and March 4, 2021, 11 340 (58%) of 19 423 patients enrolled into the RECOVERY trial were eligible to receive colchicine (ie, colchicine was available in the hospital at the time of their admission and the attending clinician was of the opinion that the patient had no known indication for or contraindication to colchicine; figure 1). 5610 (49%) patients were randomly assigned to the colchicine group and 5730 (51%) were randomly assigned to the usual care group (36 [<1%] patients were randomly assigned in Nepal and Indonesia). The mean age of study participants was 63·4 years (SD 13·8), and the median time since symptom onset was 9 days (IQR 6–12; table 1; appendix p 44) in both groups. At randomisation, 10 603 (94%) of patients were receiving corticosteroids.

**Table 1:** Baseline characteristics

		**Colchicine group (n=5610)**	**Usual care group (n=5730)**
Age, years		63·3 (13·8)	63·5 (13·7)
Age groups, years			
	<70	3806 (68%)	3850 (67%)
	70 to <80	1139 (20%)	1227 (21%)
	≥80	665 (12%)	653 (11%)
Sex			
	Male	3897 (69%)	4012 (70%)
	Female	1713 (31%)	1718 (30%)
Ethnicity			
	White	4344 (77%)	4383 (76%)
	Black, Asian, and minority ethnic	758 (14%)	813 (14%)
	Unknown	508 (9%)	534 (9%)
Number of days since symptom onset		9 (6–12)	9 (6–12)
Number of days since admission to hospital		2 (1–3)	2 (1–3)
Respiratory support received			
	None or simple oxygen	3815 (68%)	3962 (69%)
	Non-invasive ventilation	1527 (27%)	1507 (26%)
	Invasive mechanical ventilation	268 (5%)	261 (5%)
Laboratory measurements			
	C-reactive protein, mg/L	86 (44–145)	87 (46–144)
	Creatinine, μmol/L	78 (64–96)	78 (65–96)
Previous diseases			
	Diabetes	1426 (25%)	1470 (26%)
	Heart disease	1189 (21%)	1231 (21%)
	Chronic lung disease	1208 (22%)	1206 (21%)
	Tuberculosis	16 (<1%)	13 (<1%)
	HIV	11 (<1%)	20 (<1%)
	Severe liver disease*	0	0
	Severe kidney impairment†	170 (3%)	166 (3%)
	Any of the above	2880 (51%)	2963 (52%)
Use of corticosteroids			
	Yes	5243 (93%)	5360 (94%)
	No	363 (6%)	365 (6%)
	Missing	4 (<1%)	5 (<1%)
Use of remdesivir		1235 (22%)	1251 (22%)
SARS-CoV-2 PCR test result			
	Positive	5456 (97%)	5553 (97%)
	Negative	57 (1%)	58 (1%)
	Unknown	97 (2%)	119 (2%)

Data are mean (SD), n (%), or median (IQR). No children or pregnant women were randomised.

* Defined as requiring ongoing specialist care.

† Defined as estimated glomerular filtration rate less than 30 mL/min per 1·73 m^2^.

The follow-up form was completed for 5510 (98%) patients in the colchicine group and 5605 (98%) patients in the usual care group. Of the patients with a completed follow-up form, 5122 (93%) in the colchicine group received at least one dose of colchicine. Of those assigned to the colchicine group who received at least one dose, 3823 (75%) received all (or nearly all, missing at most 1 day of treatment) of their scheduled doses during their hospital stay; 4576 (90%) received at least half of their scheduled doses (figure 1; appendix p 45). The median duration of treatment with colchicine was 6 days (IQR 3–9). Use of other treatments for COVID-19 was similar between patients in both groups, with 9675 (87%) receiving a corticosteroid, 2542 (23%) receiving remdesivir, and 1485 (13%) receiving tocilizumab or sarilumab (appendix p 45).

**Figure 2:**
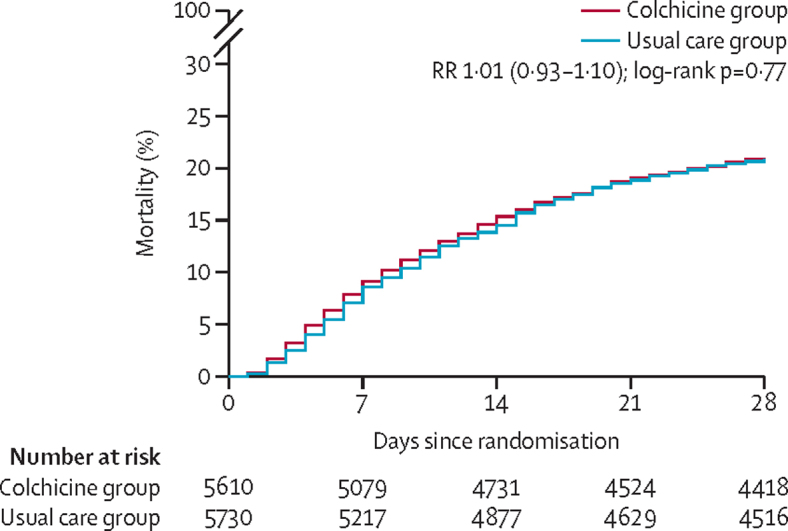
Effect of allocation to colchicine on 28-day mortality

**Figure 3:**
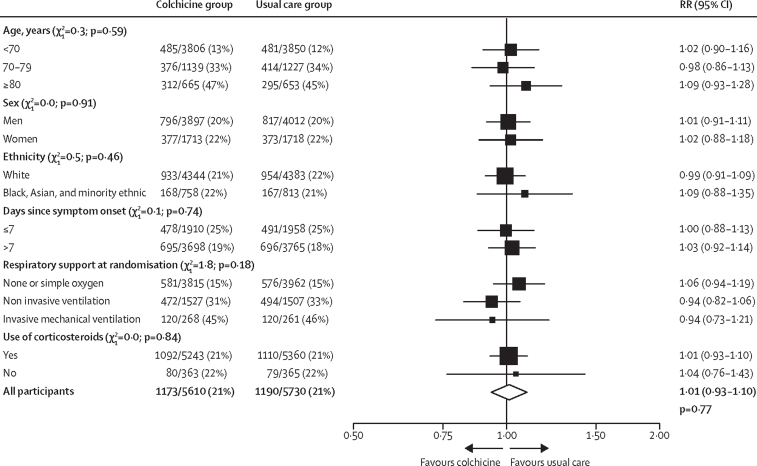
Effect of allocation to colchicine on 28-day mortality by baseline characteristics Ethnicity, days since onset, and use of corticosteroids subgroups exclude those with missing data, but these patients are included in the overall summary.

Primary outcome data are known for 11282 (99%) of the randomly assigned patients. There was no significant difference in the proportion of patients who died within 28-days between the two groups (1173 [21%] patients in the colchicine group *vs* 1190 [21%] patients in the usual care group; rate ratio 1·01 [95% CI 0·93–1·10]; p=0·77; figure 2). We observed similar results across all prespecified subgroups (figure 3) and in an exploratory analysis by baseline CRP concentration (appendix p 49). In an exploratory analysis restricted to the 11 009 (97%) patients with a positive SARS-CoV-2 test result, the result was virtually identical to the overall result (rate ratio 1·02 [0·94–1·10]; p=0·70).

The median time to discharge from hospital alive was 10 days (IQR 5 to >28) in both groups, and there was no significant difference in the probability of being discharged alive within 28 days between the two groups (3901 [70%] patients in the colchicine group and 4032 (70%) usual care group; rate ratio 0·98 [95% CI 0·94 to 1·03]; p=0·44; table 2). Of the patients not on invasive mechanical ventilation at baseline, the number of patients progressing to the prespecified composite secondary outcome of invasive mechanical ventilation or death was similar in both groups (1344 [25%] in the colchicine group *vs* 1343 [25%] patients in the usual care group; risk ratio 1·02 [95% CI 0·96 to 1·09]; p=0·47). Similar results were seen in all prespecified subgroups of patients (appendix pp 50–51).

**Table 2:** Effect of allocation to colchicine on key study outcomes

		**Colchicine group (n=5610)**	**Usual care group (n=5730)**	**RR (95% CI)**	**p value**
**Primary outcome**					
28-day mortality		1173 (21%)	1190 (21%)	1·01 (0·93–1·10)	0·77
**Secondary outcomes**					
Median time to being discharged alive, days		10 (5 to >28)	10 (5 to >28)	..	..
Discharged from hospital within 28 days		3901 (70%)	4032 (70%)	0·98 (0·94–1·03)	0·44
Receipt of invasive mechanical ventilation or death*		1344/5342 (25%)	1343/5469 (25%)	1·02 (0·96–1·09)	0·47
	Invasive mechanical ventilation	600/5342 (11%)	591/5469 (11%)	1·04 (0·93–1·16)	0·48
	Death	1053/5342 (20%)	1070/5469 (20%)	1·01 (0·93–1·09)	0·85
**Prespecified subsidiary clinical outcomes**					
Receipt of ventilation†		852/3815 (22%)	941/3962 (24%)	0·94 (0·87–1·02)	0·14
Non-invasive ventilation		818/3815 (21%)	904/3962 (23%)	0·94 (0·86–1·02)	0·14
Invasive mechanical ventilation		259/3815 (7%)	228/3962 (6%)	1·18 (0·99–1·40)	0·06
Successful cessation of invasive mechanical ventilation‡		88/268 (33%)	81/261 (31%)	1·01 (0·75–1·37)	0·93
Use of haemodialysis or haemofiltration§		212/5570 (4%)	203/5683 (4%)	1·07 (0·88–1·29)	0·51

Data are n (%) or n/N (%). RR=rate ratio for the outcomes of 28-day mortality, hospital discharge, and successful cessation of invasive mechanical ventilation, and risk ratio for other outcomes.

* Analyses exclude those on invasive mechanical ventilation at randomisation.

† Analyses exclude those on any form of ventilation at randomisation.

‡ Analyses restricted to those on invasive mechanical ventilation at randomisation.

§ Analyses exclude those on haemodialysis or haemofiltration at randomisation.

We found no significant differences in the prespecified subsidiary clinical outcomes of cause-specific mortality (appendix p 46), use of ventilation, successful cessation of invasive mechanical ventilation, or need for haemodialysis or haemofiltration (table 2). The incidence of new cardiac arrhythmias, bleeding events, and thrombotic events was also similar in the two groups (appendix p 47). There were two reports of a serious adverse reaction believed related to colchicine: one patient had severe acute kidney injury and one had rhabdomyolysis.

## Discussion

In this large, randomised trial involving more than 11 000 patients from three countries and more than 2000 deaths, use of colchicine was not associated with a reduction in mortality, duration of hospitalisation, or the risk of being ventilated or dying for those not on ventilation at baseline. These results were consistent across the prespecified subgroups of age, sex, ethnicity, duration of symptoms before randomisation, level of respiratory support at randomisation, and use of corticosteroids.

The benefit of dexamethasone in patients with COVID-19 requiring respiratory support shows the importance of inflammation in this patient group and colchicine was proposed as a treatment for COVID-19 based on its anti-inflammatory activity.^25^ However, in this large, well powered trial, we found no evidence of a benefit from colchicine, which suggests that the anti-inflammatory properties of colchicine are either insufficient to produce a meaningful reduction in mortality risk or are not affecting the relevant inflammatory pathways in moderate-to-severe COVID-19. The protocol included a maximum of 10 days of treatment with colchicine. It is possible that a longer duration of therapy might have provided benefit, but most participants had stopped colchicine before 10 days either because of death, discharge from hospital, or at the discretion of the treating clinician. Although, most patients in this study received concomitant corticosteroid therapy, we saw no evidence that colchicine was beneficial in those patients not receiving a corticosteroid.

Strengths of this trial included that it was randomised, had a large sample size, broad eligibility criteria, was international, and more than 99% of patients were followed up for the primary outcome of all-cause mortality. However, there are some limitations. Detailed information on laboratory markers of inflammation and immune response and information on radiological features was not collected; therefore, it is not possible to assess if the effect of treatment varied between such subgroups of patients. Although this randomised trial is open-label, the outcomes are unambiguous and were ascertained without bias through linkage to routine health records.

Three other randomised controlled trials have assessed the efficacy of colchicine for the treatment of patients hospitalised with COVID-19.^18^, ^19^, ^20^ A two day shorter duration of hospitalisation was reported in a trial of 100 patients with laboratory confirmed SARS-CoV-2 infection and pulmonary involvement—confirmed by CT—who were randomly assigned to receive either hydroxychloroquine plus colchicine or hydroxychloroquine plus placebo.^18^ A second trial reported a reduced duration of hospitalisation and oxygen therapy in 36 patients hospitalised with COVID-19 allocated colchicine compared with 36 patients allocated usual care, which included hydroxychloroquine, azithromycin, and methylprednisolone.^19^ Finally, the GRECCO-19 trial^20^ reported a lower rate of clinical deterioration in 55 patients randomly assigned to receive colchicine compared with 50 patients randomly assigned to receive usual care, which did not include corticosteroids.^20^ The total number of patients in all three of these trials combined was 285, with seven deaths during the follow-up period, meaning that, even combined, these three studies are not able to reliably assess the effects of colchicine on mortality. By contrast, the RECOVERY trial, with more than 11 000 participants and more than 2000 deaths, had excellent power to detect modest treatment benefits; none were observed.

The RECOVERY trial only studied patients who had been hospitalised with COVID-19; therefore, we are not able to provide any evidence on the safety and efficacy of colchicine used in other patient groups. In the COLCORONA trial^26^ of 4488 non-hospitalised patients with laboratory confirmed or clinically suspected COVID-19, fewer patients in the colchicine group died or were hospitalised within 30 days of randomisation than in the placebo group. However, the trial was stopped before the scheduled sample size had been fully enrolled due to logistical reasons, and the result was not statistically significant (odds ratio 0·79 [95% CI 0·61–1·03]; p=0·081).^26^ Thus, the role of colchicine in treatment of COVID-19 in patients not requiring hospitalisation remains uncertain. Future trials in this setting are ongoing.^27^

In summary, the results of this large, randomised trial do not support the use of colchicine in adults hospitalised with COVID-19.

Correspondence to: Prof Peter W Horby and Prof Martin J Landray, RECOVERY Central Coordinating Office, Oxford OX3 7LF, United Kingdom. recoverytrial@ndph.ox.ac.uk

## Data sharing

The protocol, consent form, statistical analysis plan, definition and derivation of clinical characteristics and outcomes, training materials, regulatory documents, and other relevant study materials are available online. As described in the protocol (appendix 2), the trial Steering Committee will facilitate the use of the study data and approval will not be unreasonably withheld. Deidentified participant data will be made available to bona fide researchers registered with an appropriate institution within 3 months of publication. However, the Steering Committee will need to be satisfied that any proposed publication is of high quality, honours the commitments made to the study participants in the consent documentation and ethical approvals, and is compliant with relevant legal and regulatory requirements (eg, relating to data protection and privacy). The Steering Committee will have the right to review and comment on any draft manuscripts prior to publication. Data will be made available in line with the policy and procedures described online. Those wishing to request access should complete the online form and sent to data.access@ndph.ox.ac.uk

## Declaration of interests

We declare no competing interests.
